# Antidiabetic activity of *Solanum torvum* fruit extract in streptozotocin-induced diabetic rats

**DOI:** 10.3389/fnut.2022.987552

**Published:** 2022-10-28

**Authors:** Namani Satyanarayana, Suresh V. Chinni, Ramachawolran Gobinath, Paripelli Sunitha, Akula Uma Sankar, Bala Sundaram Muthuvenkatachalam

**Affiliations:** ^1^Department of Anatomy, Saint James School of Medicine, Saint Vincent, Saint Vincent and the Grenadines; ^2^Department of Biochemistry, Faculty of Medicine, Bioscience, and Nursing, MAHSA University, Jenjarom, Selangor, Malaysia; ^3^Department of Periodontics, Saveetha Dental College and Hospitals, Saveetha Institute of Medical and Technical Sciences, Chennai, India; ^4^Department of Foundation, RCSI and UCD Malaysia Campus, Georgetown, Pulau Pinang, Malaysia; ^5^Department of Physiology, Saint James School of Medicine, Saint Vincent, Saint Vincent and the Grenadines; ^6^Faculty of Medicine, Biochemistry Unit, AIMST University, Bedong, Kedah, Malaysia

**Keywords:** antidiabetic activity, streptozotocin, lipid profile, hepatoprotective activity, gene expression, *Solanum torvum*

## Abstract

**Background:**

*Solanum torvum* Swartz, a medicinal plant belonging to the family Solanaceae, is an important medicinal plant widely distributed throughout the world and used as medicine to treat diabetes, hypertension, tooth decay, and reproductive problems in traditional systems of medicine around the world including Malaysia. The objective of this study was to investigate hypoglycemic, antilipidemic, and hepatoprotective activities, histopathology of the pancreas, and specific glucose regulating gene expression of the ethanolic extract of *S. torvum* fruit in streptozotocin-induced diabetic Sprague–Dawley rats.

**Materials and methods:**

Acute toxicity study was done according to OECD-423 guidelines. Diabetes was induced by intraperitoneal (i.p.) injection of streptozotocin (55 mg/kg) in male Sprague–Dawley rats. Experimental diabetic rats were divided into six different groups; normal, diabetic control, and glibenclamide at 6 mg/kg body weight, and the other three groups of animals were treated with oral administration of ethanolic extract of *S. torvum* fruit at 120, 160, and 200 mg/kg for 28 days. The effect of ethanolic extract of *S. torvum* fruit on body weight, blood glucose, lipid profile, liver enzymes, histopathology of pancreas, and gene expression of glucose transporter 2 (slc2a2), and phosphoenolpyruvate carboxykinase (PCK1) was determined by RT-PCR.

**Results:**

Acute toxicity studies showed LD_50_ of ethanolic extract of *S. torvum* fruit to be at the dose of 1600 mg/kg body weight. Blood glucose, total cholesterol, triglycerides, low-density lipoproteins, very low-density lipoproteins, serum alanine aminotransferase, and aspartate aminotransferase were significantly reduced, whereas high-density lipoproteins were significantly increased in *S. torvum* fruit (200 mg/kg)-treated rats. Histopathological study of the pancreas showed an increase in number, size, and regeneration of β-cell of islets of Langerhans. Gene expression studies revealed the lower expression of *slc2a2* and *PCK1* in treated animals when compared to diabetic control.

**Conclusion:**

Ethanolic extract of *S. torvum* fruits showed hypoglycemic, hypolipidemic, and hepatoprotective activity in streptozocin-induced diabetic rats. Histopathological studies revealed regeneration of β cells of islets of Langerhans. Gene expression studies indicated lower expression of *slc2a2* and *PCK1* in treated animals when compared to diabetic control, indicating that the treated animals prefer the gluconeogenesis pathway.

## Introduction

Diabetes mellitus is a chronic and non-communicable leading public health problem. Diabetes mellitus is a metabolic disorder of carbohydrate due to insulin deficiency resulting from dysfunction of pancreatic beta cells. Over the past decade, diabetes prevalence has risen faster in low- and middle-income countries compared to high-income countries. One of the risk factors being the overweight with possible complications include heart attack, stroke, kidney failure, leg amputation, vision loss, and nerve damage. In addition, during pregnancy, poorly controlled diabetes increases the risk of fetal death and other complications (1).

Diabetes is a potential public health problem in Malaysia and according to the National Health Survey the Ministry of Health, Malaysia reported that the prevalence of diabetes was 13.80% for men and 14.54% for women. In terms of the main ethnic groups, the most common is in the Indian’s subpopulation (25.10%), followed by the Malays (15.25%), Chinese (12.87%), Bumiputera (8.62%), and others (6.91%) (2). About two to three decades ago, most of the drugs were obtained from natural sources. Herbal plants have been used for the treatment of various disorders with no sound scientific knowledge on its function, phyto-chemistry, and adverse effects (3). Thus, the focus of this study is to establish scientific basis of antidiabetic effect of ethanolic fruit extract of *Solanum torvum* fruit through biochemical, histopathological, and molecular evidence.

Medicinal plants play an important role in both preventive and curative medicinal preparations for human beings. Herbal medicines are the only affordable source of healthcare, especially for the poorest patients (4). Furthermore, herbal medicines are gaining popularity both in developing and developed countries due to their safety, efficacy, quality, very low adverse effects, and easy availability. Some of the currently available drugs such as aspirin, digitalis, quinine (anti-malarial), vincristine, and vinblastine (anti-cancerous) were derived from the plant sources. Plant-derived phytochemicals have beneficial effect against diabetes, microorganism, inflammation, cardiovascular diseases, blood disorders, cerebral disorders, immune system, oxidative stress, reproductive disorder, and cancer chemotherapy (5). According to the World Health Organization (WHO), more than 21,000 plants are used for medicinal purposes in the world (6). Ethnobotanical information reports about 800 plants which possess antidiabetic potential (7).

Despite the introduction of many new antidiabetic drugs from natural and synthetic sources, diabetes and its secondary complications continue to be a major medical problem. Many indigenous medicinal plants have been found to be useful to successfully manage diabetes. One of the great advantages of medicinal plants is that these are readily available and have very low adverse effects (8). Even though plant sources are potential antidiabetic drugs, they have not gained sufficient momentum among the scientific community. In recent times, it is understood that a proper nutritional regulation with appropriate herbs in our diet shall help to reduce the incidence of diabetes (9). *S. torvum* fruit is also infrequently used by a section of a population as a vegetable. From the literature, it is evident that it is used against various diseases because of its rich phytochemical contents. Furthermore, not much study has been directed toward its potential as antidiabetic activity particularly with the *S. torvum* found locally in Malaysia. Therefore, the aim of this study was to find out the scientific basis of the use of *S. torvum* fruits in the management of diabetes used by traditional practitioners using 70% ethanol extracts on **streptozotocin**-induced diabetic rats.

## Collection of plant material

*Solanum torvum* fruits were collected from the local market, Bedong, Sungai Petani, Kedah, Malaysia. The fruits were authenticated by a plant biologist, AIMST University, Malaysia. The herbarium with voucher specimen (specimen no: 13455) was deposited with the Faculty of Applied Sciences, AIMST University, Malaysia. The fruits were washed with distilled water and dried under shade, powdered finely using heavy duty blender (Waring commercial, USA), and stored at 4°C until further use.

## Chemicals and instruments

Streptozotocin (Sigma Chemical Company, St Louis, MO, United States) was used to induce diabetes in rats, and glibenclamide (Hoechst Pharmaceuticals, Mumbai, India) was used as a standard hypoglycemic drug. Diethyl ether was used as anesthetic, and ethanol (BDH Ltd., Mumbai, India) and distilled water were used for extraction of the plant materials. Glucometer, 3,5-dinitrosalicylic acid (DNSA) (Sigma-Aldrich Co., St. Louis, MO, USA), and Accu-Check^®^ Active glucometer test strips (Hoffman-La Roche Ltd., Basel, Switzerland) were used to carry out the experiment. All other used reagents were of analytical grade.

## Preparation of plant crude extract

The finely powdered *S. torvum* fruits (100 gm) were mixed with ethanol (500 ml) for the preparation of ethanolic extracts by cold maceration process for a period of 72 h (10). Ethanolic extracts were prepared by maceration process and concentrated using rotary evaporator (Eyela Rotary evaporator N-1000, Japan). Then, the extracts were dried in an oven (Sanyo Microwave Oven Electric Co. Ltd., Taipei, Taiwan) at 40°C. After drying, the amount of dry extract obtained was harvested, and the dried extract was transferred into airtight bottles and stored in a refrigerator at −4°C until used. The weight of the dry extract was expressed as percentage of the total mass of dry plant matter to determine the percentage yield.

## Experimental animals

This study was carried on forty healthy adult male Sprague–Dawley (SD) rats, weighing 170–200 gm, which were obtained from Central Animal House, AIMST University, Bedong, Malaysia. The animals were housed in large spacious poly-acrylic cages at an ambient room temperature with 12-h light/12-h dark cycle under standard laboratory and environmental conditions. The animals were free access to water and fed with standard rat feed *ad libitum*. The study was approved by AIMST University Human and Animal Ethics Committee (AUHAEC8/FAS, 2012).

## Acute oral toxicity study

The determination of LD_50_ for the extract was carried out as per the guidelines of OECD-423 (11). In this toxicity study, Sprague–Dawley (SD) male rats were weighed 170–200 gm g (*n* = 3) and selected by random sampling technique. The animals were fasted for 4 h prior to the experiment and maintained under standard conditions of temperature (22 ± 1°C) and humidity (55 ± 3°C). The rats were allowed to free access to water. The *S. torvum* fruit extract was dissolved in distilled water and administered orally by gavage with the initial doses. The general behavior of the experimental rats was observed continuously over a period of 24 h for any signs of toxicity and the latency of death (12).

## Induction of diabetes in Sprague–Dawley rats

In this study, 16-week-old normal (fasting blood glucose level of 90–110 mg/dl) rats were used. A single dose of intraperitoneal injection of STZ (55 mg/kg/i.p) (Sigma, St. Louis, MO, USA) dissolved in 0.1M citrate buffer (pH 4.5) was used to induce diabetes in overnight fasted male SD rats weighing 170–200 gm. Rats were allowed to free access to 10% glucose water to prevent hypoglycemia. After 72 h, the rats were checked for the blood glucose level from the tail vein using glucometer (Accu-check, Roche Diagnostic, Indianapolis, IND, United states). Only the rats with fasting blood glucose levels ≥ 250 mg/dl were considered as diabetic-induced rats and included in this study.

## Animal experimental design

No mortality was observed at the acute oral dose of 1600 mg/per kg body weight by the oral route. The medial lethal dose following oral administration was 1800 mg/per kg body weight. For the selection of doses, 7.5, 10, and 12.5% of 1600 mg/kg body weight were used as concentration of doses for *S. torvum* (13). Six normal healthy rats were chosen randomly for the control group. Thirty diabetic-induced rats were selected, and six rats were randomly assigned for each group for the study.

Group I: Control rats orally administered with distilled water.
Group II: Streptozotocin-induced diabetic rats administered orally with distilled water.
Group III: Streptozotocin-induced diabetic rats administered orally with *Glibenclamide* (5 mg/kg) dissolved in distilled water.
Group IV: Streptozotocin-induced diabetic rats administered orally with ethanolic extract of *S. torvum* fruit (120 mg/kg) dissolved in distilled water.
Group V: Streptozotocin-induced diabetic rats administered orally with ethanolic extract of *S. torvum* fruit (160 mg/kg) dissolved in distilled water.
Group VI: Streptozotocin-induced diabetic rats administered orally with ethanolic extract of *S. torvum* fruit (200 mg/kg) dissolved in distilled water.

All the treatments were started on the fourth day after STZ injection and once a day continued for 28 days.

## Biochemical analysis

After 28 days of treatment, blood samples were collected from the retro-orbital plexus, and blood glucose levels were estimated using glucometer (Accu-check, Roche Diagnostic, Indianapolis, IND, United states) before sacrificing the rats (14). The remaining blood was centrifuged at 3000 rpm for 5 min. Serum was collected immediately and stored at −70°C until the analysis of biochemical parameters. The serum was used for the estimation of biochemical parameters such as lipid profile [total serum cholesterol, serum triglyceride, high-density lipoprotein (HDL), low-density lipoprotein (LDL), and very low-density lipoprotein (VLDL)] and liver function tests [serum glutamate oxaloacetate transaminase (ALT) and serum glutamate pyruvate transaminase (AST)]. These biochemical parameters were measured using Reflectron plus (Roche, Germany) (15).

## Histopathological study

The animals were sacrificed by anesthetized with diethyl ether and cervical dislocation; after the dissection of pancreas, it was quickly washed in ice-cold isotonic saline and blotted on ash-free filter paper. Then, the organ was processed immediately for the histopathological studies. The tissue processing procedure includes fixation, dehydration, clearing, and embedding of the materials in wax and block making, microtomy, and finally stained by hematoxylin and eosin (H and E) and mounting the sections on a slide (10). All tissue processing were done in Leica TP1020 Semi-enclosed Bench top Automatic Tissue Processor. The tissue-embedded block was used for sectioning using rotary microtome to obtain a tissue section of 5 μ thickness.

## Gene expression studies

### Reverse transcription-polymerase chain reaction

The effects on the expression level of some genes involved in production, secretion, and regulation of insulin were analyzed by reverse transcription-polymerase chain reaction (RT-PCR). After 6 and 24 h treatment, total RNA from cells was extracted using GENEzol™ reagent as described by the manufacturer (Geneaid, Taipei, Taiwan). Complimentary DNA (cDNA) of the *slc2a2, PCK1*, and β*-actin* was synthesized from total RNA using specific reverse primers by reverse transcriptase enzyme (Superscript reverse transcriptase IV, Thermo Fisher Scientific, Xinjiang, China) as shown in Table 1. Complimentary DNA (cDNA) of the control gene was synthesized from HeLa total RNA using specific reverse primers by reverse transcriptase enzyme which was supplied by Thermo Fisher Scientific, Xinjiang, China. RT-PCR was performed using SYBR Premix Ex Taq technology (TaKaRa Bio Inc., Otsu, Shiga, Japan) on the Applied Biosystems StepOne RT-PCR system. PCR products were analyzed using 1.5% agarose gel electrophoresis under UV transilluminator (BioRad, USA).

**TABLE 1:** Gene name, amplicon size (bp), and forward and reverse primer sequences that were used in polymerase chain reaction (PCR).

Gene name	Amplicon size in bases	Annealing temp	Forward primer 5′-3′	Reverse primer 5′-3′
NM_031144.2 (Actb)	97	49°C	ATGGTGGGTATG GGTCAG	CAATGCCGTGTTCAATGG
NM_012879.2 (*slc2a2*)	162	50.3°C	TCTGTGCTGCTTGTGGAG	ACTGACGAAGAGGAAGATGG
NM_198780.3 (*PCK1*)	171	51.5°C	AACGTTGGCTGGCTCTC	GAACCTGGCGTTGAATGC
Control gene	353	52°C	GCTCGTCGTCGACAACGGCTC	CAAACATGATCTGGGTCATCTTCTC

## Results

The yield of crude extract was 0.8% (ethanol extract). The body weight of diabetic control group was significantly reduced as compared to the normal control group. The animals treated with glibenclamide (6 mg/kg) and *S. torvum* extract for 28 days significantly increased the body weight as compared to the diabetic control group. The body weight of normal control group was significantly increased on day 14 and 28 compared to day 0, while in the diabetic control group which was significantly decreased on day 14 and 28 compared to day 0. However, the body weight of diabetic rats treated with ethanol extract of *S. torvum* fruit in different doses (120, 160, and 200 mg/kg) was significantly increased (Table 2).

**TABLE 2:** Effect of ethanoic extract of *Solanum torvum* fruits on body weight of streptozotocin (STZ)-induced diabetic rats.

Groups	Day 0	Day 14	Day 28
Group–I	181.67 ± 1.63^a^	192.67 ± 3.26^b^	202.50 ± 3.78^b^
Group–II	205.00 ± 8.98^c^	176.33 ± 8.73^a^	154.67 ± 10.32^a^
Group–III	189.33 ± 4.84^ab^	204.50 ± 6.97^cd^	221.33 ± 2.65^c^
Group–IV	184.33 ± 2.87^a^	197.00 ± 3.68^bc^	206.50 ± 4.37^b^
Group–V	192.83 ± 9.60^b^	204.17 ± 9.17^cd^	219.17 ± 3.54^c^
Group–VI	187.33 ± 6.50^ab^	206.33 ± 6.37^d^	227.67 ± 1.86^d^

The values are expressed as mean ± SD (*n* = 6). Values in the column having similar superscripts are not statistically different (*P* < 0.05) (one-way ANOVA followed by Duncan’s multiple comparison test).

The results indicated that the fasting blood glucose levels during the experimental period (day 0–28) were significantly higher in diabetic control group as compared with the normal control group. Significantly decreased blood glucose levels were observed in glibenclamide group (6 mg/kg) from day 0 to 28 (101.83 ± 3.76 mg/dl) when compared to diabetic control group (280.83 ± 7.026 mg/dl). Similarly, blood glucose levels were significantly reduced in ethanolic extract of *S. torvum* fruit-treated rats (120, 160, and 200 mg/kg) compared to diabetic control group. Statistically lowest blood glucose level was observed in glibenclamide group (101.83 ± 3.76 mg/dl). Significantly, decreased blood glucose levels were observed in *S. torvum* (200 mg/kg)-treated group compared to other *S. torvum* (120 and 160 mg/kg)-treated groups. A dose-dependent effect on fasting blood glucose levels was recorded in groups IV, V, and VI (Table 3).

**TABLE 3:** Effect of ethanol extract of *Solanum torvum* fruits on fasting blood glucose level in streptozotocin (STZ)-induced diabetic Sprague–Dawley (SD) rats.

Fasting blood glucose mg/dl					

Groups	Day 0	Day 7	Day 14	Day 21	Day 28
Group–I	93.33 ± 2.582^a^	92.67 ± 4.45^a^	93.67 ± 4.32^a^	93.00 ± 1.78^a^	90.43 ± 2.36^a^
Group–II	292.00 ± 5.13^d^	278.83 ± 12.33^e^	277.00 ± 9.77^f^	280.33 ± 6.56^f^	280.83 ± 7.026^f^
Group–III	294.83 ± 5.87^d^	261.83 ± 9.17^d^	165.67 ± 9.52^b^	128.83 ± 6.85^b^	101.83 ± 3.76^b^
Group–IV	281.67 ± 9.18^bc^	257.67 ± 5.46^d^	244.17 ± 3.71^e^	232.83 ± 3.37^e^	210.00 ± 7.61^e^
Group–V	280.83 ± 9.66^b^	241.00 ± 7.72^c^	211.83 ± 19.65^d^	165.83 ± 18.35^d^	137.33 ± 6.28^d^
Group–VI	289.83 ± 6.73^cd^	205.50 ± 13.03^b^	184.00 ± 15.47^c^	141.50 ± 14.57^c^	118.16 ± 3.81^c^

The values are expressed as mean ± SD (*n* = 6). Values in the column having different superscripts are statistically different (*P* < 0.05) (One-way ANOVA followed by Duncan’s multiple comparison test).

Total cholesterol, triglycerides, low-density lipoproteins, and very low-density lipoproteins increased significantly in diabetic control group as compared with normal control. On the contrary, the level of high-density lipoproteins decreased significantly in diabetic control group compared to normal control. Serum TC, TG, LDL, and VLDL were significantly reduced, whereas HDL was significantly increased in *S. torvum* fruit (120, 160, and 200 mg/kg)-treated groups in a dose-dependent manner. The anti-hyperlipidemic effect of glibenclamide (6 mg/kg) was comparable with the treatment of 200 mg/kg body wt of ethanolic fruit extract of *S. torvum* (Table 4).

**TABLE 4:** Effect of ethanolic extract of *Solanum torvum* fruits on lipid profile in streptozotocin (STZ)-induced diabetic Sprague–Dawley (SD) rats.

Lipid profile (mg/dl)					

Groups–Treatment	Serum total cholesterol mg/dl	Triglycerides mg/dl	S.HDL mg/dl	S.LDL mg/dl	S.VLDL mg/dl
Group–I	94.31 ± 2.64^a^	84.31 ± 2.64^a^	33.10 ± 3.50^c^	44.35 ± 4.64^a^	16.85 ± 0.52^a^
Group–II	130.84 ± 5.03^e^	123.58 ± 3.24^e^	23.01 ± 1.63^a^	83.11 ± 4.86^e^	24.71 ± 0.64^e^
Group–III	106.13 ± 4.79^b^	92.30 ± 2.52^b^	36.42 ± 2.58^d^	51.25 ± 4.33^b^	18.45 ± 0.50^b^
Group–IV	124.17 ± 3.77^d^	111.18 ± 3.58^d^	29.43 ± 1.38^b^	72.51 ± 3.87^d^	22.23 ± 0.71^d^
Group–V	117.54 ± 3.06^c^	106.23 ± 3.52^c^	31.27 ± 1.43^bc^	65.26 ± 1.99^c^	21.24 ± 0.70^c^
Group–VI	107.82 ± 3.63^b^	93.01 ± 4.12^b^	33.58 ± 2.60^c^	55.64 ± 4.33^b^	18.59 ± 0.82^b^

The values are expressed as mean ± SD (*n* = 6). Values in the column having different superscripts are statistically different (*P* < 0.05) (One-way ANOVA followed by Duncan’s multiple comparison test).

The serum alanine aminotransferase (ALT) and aspartate aminotransferase (AST) levels were increased significantly in diabetic control group compared to normal control group. There was a significant reduction of ALT and AST in glibenclamide group (6 mg/kg) compared to diabetic control group. Similarly, a dose-dependent reduction of ALT and AST was observed in *S. torvum* fruit extract-treated (120, 160, and 200 mg/kg) groups compared to diabetic control group (Table 5).

**TABLE 5:** Effect of ethanolic extract *Solanum torvum* fruits on liver enzymes in streptozotocin-induced diabetic Sprague–Dawley (SD) rats.

Liver enzymes		

Groups–Treatment	ALT (U/L)	AST (U/L)
Group–I	35.68 ± 2.62^ab^	56.63 ± 3.18^b^
Group–II	56.17 ± 3.57^d^	67.56 ± 2.63^e^
Group–III	33.83 ± 2.97^a^	52.57 ± 2.97^a^
Group–IV	42.4 ± 3.00^c^	65.54 ± 1.17^de^
Group–V	38.41 ± 1.09^b^	63.99 ± 1.21^cd^
Group–VI	35.11 ± 2.02^ab^	60.95 ± 1.41^c^

The values are expressed as mean ± SD (*n* = 6). Values in the column having different superscripts are statistically different (*P* < 0.05) (One-way ANOVA followed by Duncan’s multiple comparison test).

**FIGURE 1:**
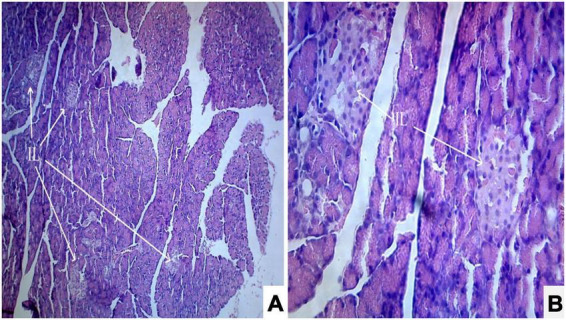
Effect of ethanolic extract *of Solanum torvum* fruit on histopathology of pancreas in streptozotocin (STZ) induced diabetic Sprague–Dawley rats (SD rats). **(A,B)** Photomicrograph of a section from pancreas (Normal control group) showed normal architecture. The acinar cells are seen to be normal. **(A)** H& E 10X; **(B)** H& E 40X. IL, islets of Langerhans cells.

**FIGURE 2:**
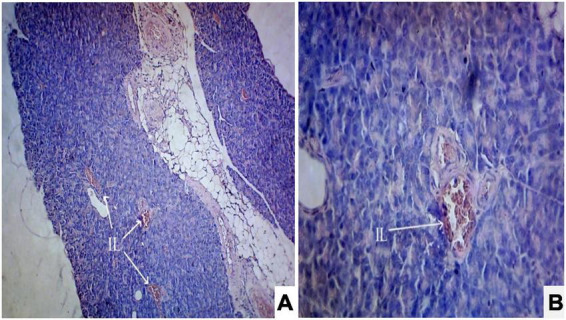
Effect of ethanolic extract *of Solanum torvum* fruit on histopathology of pancreas in streptozotocin (STZ)-induced diabetic Sprague–Dawley rats (SD rats). Photomicrograph of a STZ-induced diabetic rat section from pancreas (diabetic control group) showed severe decrease in number of islets of Langerhans cells and β-cells. The acinar cells are seen to be normal. **(A)** H& E 10X; **(B)** H& E 40X. IL, islets of Langerhans cells.

**FIGURE 3:**
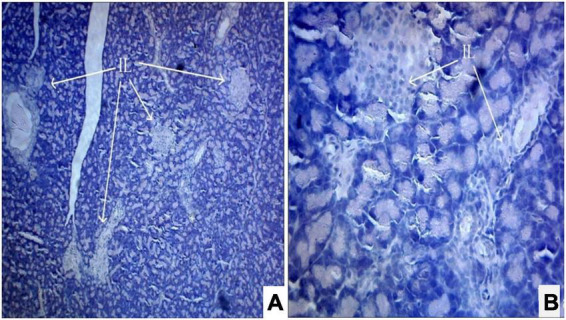
**(A,B)** Photomicrograph of pancreas from glibenclamide (6 mg/kg)-treated diabetic rat showing partial restoration of normal cellular population and size of islet cells. The acinar cells are seen to be normal. **(A)** H& E 10X; **(B)** H& E 40X. IL, islets of Langerhans cells.

Photomicrograph (Figures 1A,B) of the normal control group showed normal acini and the β-cells of islets of Langerhans. Contrastingly, an extensive cellular damage of the β-cells was observed (Figures 2A,B) in STZ-induced diabetic rats. However, the diabetic rats treated with glibenclamide (6 mg/kg) showed an increased number of regenerated β-cells (Figures 3A,B). Histopathological evidence of pancreas of animals treated with ethanolic extract of *S. torvum* fruits showed comparable regeneration of β cells comparable to that of glibenclamide-treated group.

**FIGURE 4:**
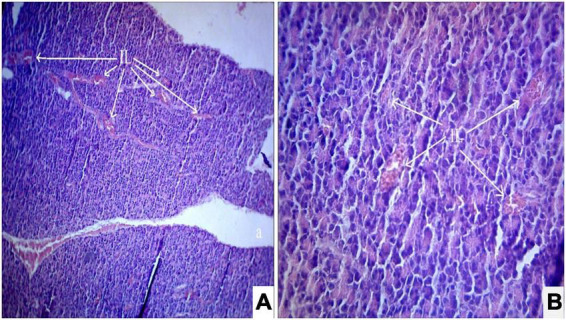
**(A,B)** Photomicrograph of a diabetic rats treated with *Solanum torvum* fruits extract (120 mg/kg/b.wt.) section from pancreas showed minimal restoration of normal cellular population and size of islet cells. The acinar cells are seen to be normal. **(A)** H& E 10X; **(B)** H& E 40X. IL, islets of Langerhans cells.

**FIGURE 5:**
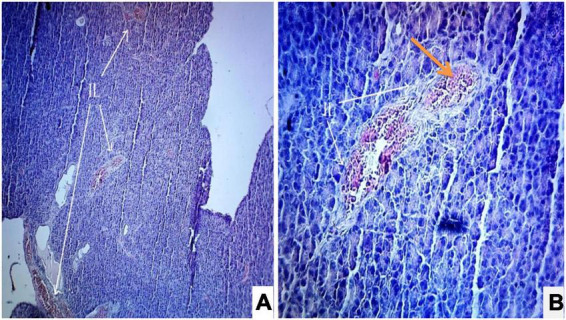
**(A,B)** Photomicrograph of a diabetic rats treated with *Solanum torvum* fruits extract (160 mg/kg/b.wt.) section from pancreas showed minimal restoration of normal cellular population and regeneration of β-cells of islets of Langerhans. The acinar cells are seen to be normal. **(A)** H& E 10X; **(B)** H& E 40X. IL, islets of Langerhans cells.

**FIGURE 6:**
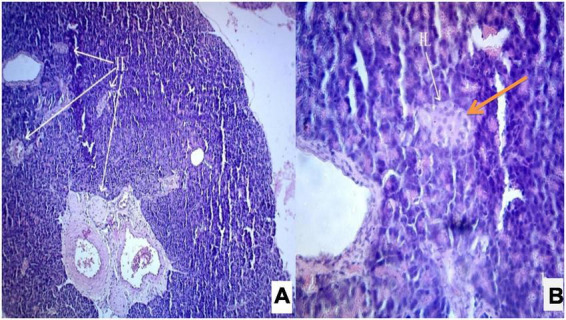
**(A,B)** Photomicrograph of a diabetic rats treated with *Solanum torvum* fruits extract (200 mg/kg/b.wt.) section from pancreas showed maximum restoration of normal cellular population and regeneration of islets of Langerhans. The acinar cells are seen to be normal. **(A)** H& E 10X; **(B)** H& E 40X. IL, islets of Langerhans cells.

Interestingly, the diabetic rats treated with ethanolic extract of *S. torvum* fruits (120, 160, and 200 mg/kg) showed a dose-dependent effect on regeneration of β-cells (Figures 4–6A,B). It is evident that the ethanolic extract of *S. torvum* fruits at 200 mg/kg showed pronounced regeneration of β-cells as compared to 120 and 160 mg/kg.

**FIGURE 7:**
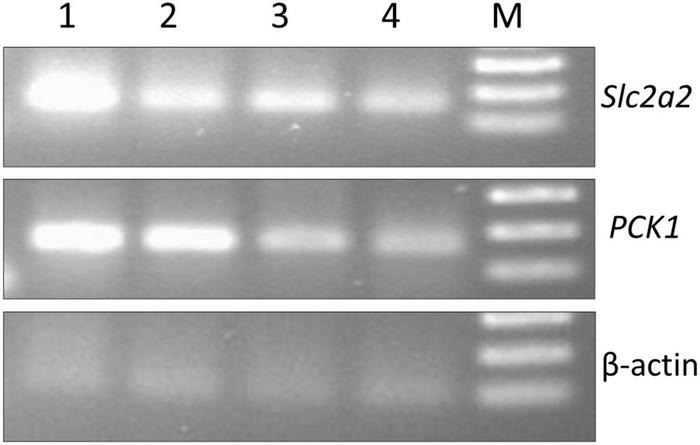
Effect of ethanolic extract of *Solanum torvum* fruit on glucose metabolism regulating gene (slc2a, PCK1, and β actin) expression study by reverse transcription-polymerase chain reaction (RT-PCR). NC, negative control; PC, positive control (genomic DNA); IC, internal control; 1, normal control group; 2, diabetic control group; 3, glibenclamide-treated group; 4, *S. torvum*-treated group; M, 100 bp DNA ladder (Thermo Fisher Scientific, Xinjiang, China).

In the present investigation, the molecular mechanism of *S. torvum* action as a hypoglycemic agent was studied by analyzing the expression levels of GLUT-2 and PCK1 genes in normal, diabetic, and diabetic-treated rats using RT-PCR assay. Significant decrease in GLUT-2 gene expression was observed in STZ-induced diabetic group compared to normal rats probably due to insulin deficiency (Figure 7). After administering *S. torvum* ethanolic fruit extract, the diabetic rats showed lower expression of GLUT-2 gene despite of decreased blood glucose level.

## Discussion

Diabetes mellitus is a disorder in which the body tissues failed to utilize the glucose which leads to increased utilization of proteins responsible for reduction in body weight (16). It has been suggested that an increase in the body weight of *S. torvum* fruit extract-treated rats might be due to an enhancement in glycemic control and increased synthesis of structural protein (17). This may be achieved *via* the inhibition of hepatic gluconeogenesis and glucose output from the liver, which is accompanied by the suppression of lipolysis in adipose tissue (18). Abu-Odeh and Talib (19) suggested that the possible mechanism for body weight gain in plant extract-treated rats may be due to extra pancreatic action which might have contributed to the increased utilization of glucose by the tissues.

Diabetes is a metabolic disorder caused by impaired metabolism of carbohydrates, proteins, and lipids predisposing to hyperglycemia (20). Glycemic control is the main target of the treatment to prevent micro- and macrovascular and neurological complications of diabetes (21). The medicinal plants are widely used as a prophylaxis and for curing of human diseases due to the presence of phytochemicals such as flavonoids and phenols in *S. torvum* fruit extract (22).

Many research reports showed that medicinal plants that possess hypoglycemic activity act through various mechanisms include improvement of insulin sensitivity of target cells, augmenting insulin secretion and stimulating the regeneration of β-cells of islets of Langerhans in pancreas (23). Several authors reported that the presence of flavonoids, steroids, terpenoids, and phenols is responsible for antidiabetic activity (24). Flavonoids have also been known to regenerate the damaged beta cells in alloxan-induced diabetic rats and act as insulin secretagogues (25).

The potential therapeutic use of polyhydroxylated alkaloids in the treatment of type 2 diabetes due to their ability to inhibit maltase-glucoamylase has been reported (26). In this study, the marked reduction in blood glucose levels may be due to regeneration of pancreatic beta cells leading to an increased secretion of insulin in *S. torvum*-treated groups. High-performance liquid chromatography revealed the presence of quercetin (flavonoid) in *S. torvum* fruit extract. It has been shown that quercetin possesses antidiabetic activity in streptozotocin-induced diabetic rats through regeneration of pancreatic islets which increases insulin secretion (27). This histological evidence may also account for the hypoglycemic activity of the *S. torvum* fruit extract. Biologically active, naturally occurring phytochemical compounds found in plants provide health benefit for humans (28).

Oxidative stress is one of the contributing factor in the pathogenesis of diabetes. Diabetes, by itself, increases the production of tissue damaging reactive oxygen species (ROS) by glucose auto-oxidation that inhibits enzymatic protein glycosylation (29). The antioxidant enzyme levels are affected by diabetes, which further increase oxidative stress (30).

Hyperglycemia and hyperlipidemia are the main causes of oxidative stress in type 2 diabetes. Reactive oxygen species (ROS) formed in this process triggers tissue damage and has been shown to affect the two major mechanisms failing during diabetes: insulin resistance and insulin secretion (31). In diabetes, tissue damage is considered to be mediated by free radicals by attacking membranes through peroxidation of unsaturated fatty acids which lead to extensive membrane damage and dysfunction (32). Decreased lipid peroxidation and improved antioxidant condition could be one of the mechanisms to prevent complications of diabetes (33).

Poongothai et al. (34) reported that methanolic extract *Solanum xanthocarpum* leaves at a dose of 200 mg/kg significantly reduced the blood glucose level and increased the serum insulin level in alloxan-induced diabetic rats. Saponins at 1.3 mg/100 g from *Solanum anguivi* fruit strongly inhibited lipid peroxidation and increased the levels of antioxidant enzymes, thus preventing hyperglycemia-induced oxidative stress (35). Diabetic rats treated with *Solanum lycocarpum* fruit extract at 1000 mg/kg resulted in reduced levels of blood glucose and also reduced food and water intake (36).

Two varieties *Solanum melongena* such as white and graffiti showed antioxidant activity, reduced the hyperglycemia-related complications, and decreased glucose absorption in the intestine (37). Similarly, ethanolic root extract of *S. xanthocarpum* at 200 and 400 mg/kg showed antihyperglycemic activity in alloxan-induced diabetic rats (38).

A recent study by Ammulu et al. (39) indicated that *Solanum trilobatum* ethanolic leaf extract at 100 and 200 mg/kg lowered blood glucose and generation of free radicals in alloxan-induced diabetic rats. Aqueous fruit extract of *Solanum nigrum* reduced blood glucose levels and hyperglycemia-related vascular complications in STZ-induced diabetic rats (40). *Solanum pubescens* methanolic leaf extract at 300 mg/kg was reported to decrease the blood glucose levels in alloxan-induced diabetic rats (41).

Generally, the diabetes is accompanied by hyperglycemia and hyperlipidemia (42). Hypercholesterolemia and hypertriglyceridemia are major risk factors for atherosclerosis which could be prevented by hypocholesterolemic drugs (29). During diabetic condition, serum fatty acids are produced in excess and converted into phospholipids and cholesterol in liver. These two substances along with excess triglycerides formed at the same time in liver may be discharged into blood in the form of lipoproteins (43). The abnormal high concentration of serum lipids in the diabetic condition is mainly due to increase in the mobilization of free fatty acids from the peripheral fat depots, since insulin inhibits the hormone-sensitive lipase.

In diabetes, the hyperlipidemia is the consequence of the uninhibited action lipolytic hormones on the fat depots (44). During normal metabolism, insulin activates lipoprotein lipase to hydrolyze triglycerides. However, in a state of insulin deficiency, lipoprotein lipase is not activated resulting in hypertriglyceridemia (45). In accordance with this study, the possible mechanism of antidiabetic and hypolipidemic activity of *S. torvum* fruit extract is mainly due to the presence of polyphenolic compounds. In addition to this, it also possesses antioxidant property which could be beneficial to diabetes (46).

Hepatoprotective, anti-inflammatory, and antioxidant effects of herbal medicines could be attributed to the presence of a variety of phytochemicals such as tannins, kaempferol, rutin, bergapten, psoralenes, flavonoids, coumarin, and phenolic glycosides (47). STZ-induced diabetic rats treated with *S. torvum* fruit extract showed decreased levels of ALT and AST indicating the hepatoprotective effect through maintenance of functional integrity of hepatic cell membrane and restoration of liver metabolism in diabetic rats (48).

The liver enzymes ALT and AST are considered to be good biomarkers of hepatotoxicity, wherein the elevated levels of these enzymes are indicative of liver cell damage (49). In diabetic rats, an increased level of these enzymes is due to the hepatic cell damage. Furthermore, the high levels of ROS play a critical role in the inflammatory damage of liver cells (50). Earlier studies have indicated the elevated levels of AST and ALT in diabetic condition including STZ-induced diabetics in experimental animals (51). It was also observed that STZ-induced diabetic rats showed a time-dependent rise in AST and ALT levels (52). Tripathi (47) reported hepatoprotective, anti-inflammatory, and antioxidant effects could be attributed to the presence of the various phytoconstituents including tannins, kaempferol, rutin, bergapten, psoralenes, flavonoids, coumarin, and phenolic glycosides. STZ-induced diabetic rats treated with *S. torvum* fruit extract showed decreased activity of ALT and AST enzymes that might support its hepatoprotective indicating maintenance of functional integrity of hepatic cell membrane, and normalization capability of impaired liver metabolism in diabetic rats (17).

The endocrine capability of pancreas is determined by apoptosis, replication, and neogenesis of beta cells of islets of Langerhans (45). Oxidative stress plays an important role in beta cell dysfunction and apoptosis (53). Apoptosis is activated by stress factors including growth factor deprivation, cell cycle disturbance, and DNA damage, which lead to mitochondrial release of cytochrome c followed by stimulation of caspase-9, 8, 3, 6, and 7 in sequence, that promote DNA fragmentation and cell death (54).

In this context, the protective effect of some phytochemicals on pancreas has been found to be mediated through their antioxidant activity (55). Furthermore, some phytochemicals stimulate the proliferation and differentiation of progenitor cells involved in protection and regeneration of β-cells (56). Most of the plants possess natural antioxidants such as phenol and flavonoids. The regeneration of pancreas may be also attributable to the tannins in the plant extracts through their anti-inflammatory action (57). The phytochemicals and amino acids in the herbal plants are associated with regeneration of β-cells in diabetic rats (58).

Modulation in gene expression related to carbohydrate metabolism is an important component of the pathogenesis of diabetes (59). In liver tissue, carbohydrate metabolism is regulated by multiple transcription factors through insulin response (60). Glucose transport is the key step in carbohydrate metabolism which is facilitated by glucose transporters. GLUT-2 is a transmembrane carrier protein that enables passive glucose movement across cell membranes (61). GLUT-2 is principal glucose transporter among the 14 GLUT protein family (62). Antihyperglycemic effect of *S. torvum* may be through a different glucose transporter rather than GLUT-2. The histopathological evidence in the treated animals showed regeneration of beta cells which may lead to an increased insulin secretion that could induce a different glucose intake pathway than GLUT-2.

Gluconeogenesis is controlled by hormone-mediated gluconeogenic enzymes at the level of gene expression. In the liver, phosphoenolpyruvate carboxykinase (*PCK1*) catalyzes the conversion of oxaloacetate to phosphoenolpyruvate and is considered to be the major rate-controlling enzyme in the gluconeogenesis pathway from pyruvate, lactate, and alanine (63). This study revealed that the ethanolic extract of *S. torvum* fruit represses *PCK1* gene expression which limits the gluconeogenesis pathway. Deficiency of glucose in the cells triggers gluconeogenesis pathway (64). The lower expression of *PCK1* was observed in treated groups compared to normal and diabetic group suggesting decreased gluconeogenesis in treated groups. This could be due to the sufficient intake of glucose by the cells after the treatment with glibenclamide or *S. torvum* fruit extract. Most of the experimental studies suggested that increased gluconeogenesis is a main source of hyperglycemia in insulin deficiency (65). It is also suggested that insulin may inhibit *PCK1* gene transcription by activation of a possible insulin response factors (66). Thus, *S. torvum*-treated diabetic rats showed the decreased expression of *PCK1* gene possibly due to the availability of insulin.

## Conclusion

The effect of *S. torvum* fruit extract on histopathology of pancreas has showed the regeneration of β cells of islets of Langerhans which may be responsible for increased secretion of insulin resulting in hypoglycemia. *S. torvum* fruit extract (200 mg/kg)-treated rats showed decreased blood glucose level may be due to lower expression of *PCK1* gene. The *PCK1* genes regulate phosphoenolpyruvate carboxykinase enzyme activity in the gluconeogenesis pathway. Thus, *S. torvum* fruits can be a good candidate for novel phytomedicine that can be used to treat several diseases.

## Data availability statement

The raw data supporting the conclusions of this article will be made available by the authors, without undue reservation.

## Ethics statement

This animal study was reviewed and approved by AIMST University Animal Ethics Committee.

## Author contributions

NS and SC have equally contributed to design and conceived the study. NS, SC, and PS carried out the experiments, conducted the data analysis, and interpreted the data. NS has procured the Institutional Ethical Approval for the study. All authors have drafted, edited, and reviewed the manuscript and given their consent for submission.
